# Role modeling of safety-leadership behaviors in the construction industry: A two-wave longitudinal study

**DOI:** 10.3233/WOR-230031

**Published:** 2024-02-07

**Authors:** Pernilla Larsman, Amanda Ulfdotter Samuelsson, Christine Räisänen, Max Rapp Ricciardi, Martin Grill

**Affiliations:** a Department of Psychology, University of Gothenburg, Gothenburg, Sweden; b Architecture and Civil Engineering, Chalmers University, Gothenburg, Sweden

**Keywords:** Occupational health, occupational accident, industrial psychology, social learning, feedback, operant conditioning, questionnaire

## Abstract

**BACKGROUND:**

The construction industry is heavily affected by occupational accidents, and it is important to investigate how leadership behaviors promoting safety on construction sites are fostered among construction-site managers.

**OBJECTIVE:**

The overall aim of this study was to investigate how safety-leadership behaviors can be developed in the construction industry, specifically focusing on managerial role modeling.

**METHODS:**

A two-wave longitudinal cohort study with approximately four months between measurement occasions was conducted among construction-site supervisors in Sweden (*n* = 51). Supervisors’ ratings of their site managers’ and their own generic and safety-specific contingent reward (CR) leadership behaviors were obtained by means of questionnaires. Cross-lagged panel models were tested within a path model framework to test the hypothesis that site managers’ leadership behaviors prospectively influence supervisors’ leadership behaviors.

**RESULTS:**

Site managers’ CR behaviors prospectively influenced supervisors’ CR behaviors, both generic CR behaviors (β= 0.29, *p* = 0.01) and safety-specific CR behaviors (β= 0.22, *p* = 0.04). For safety-specific CR behaviors, a reversed effect (β= 0.26, *p* = 0.03) was also found, implying that supervisors’ behaviors prospectively influenced site managers’ behaviors.

**CONCLUSION:**

Site managers act as role models for supervisors when it comes to developing safety-leadership behaviors on construction sites. The results also indicate that site managers are influenced by their subordinate supervisors’ safety-leadership behaviors. Hence, there seems to be reciprocal interaction between site managers and supervisors in which they influence each other and together shape safety-leadership practices at their construction sites.

## 1 Introduction

Occupational accidents are common in the construction industry, and in 2019 it accounted for over 20% of all occupational fatalities in Europe [1, 2]. Swedish statistics for 2020 indicate that work-related accidents resulting in sick leave are common in the construction industry, affecting about 10 per 1000 employees among men and about 5 per 1000 employees among women [3]. Also, work-related diseases are common, affecting about 1 per 1000 employees among both men and women [3]. A recent cohort study of male Swedish construction workers [4] found that risks of disability benefits at 60–64 years of age were reduced among workers who changed occupation from construction work to work in other industries.

Leadership behaviors on construction sites, both generic [e.g., 5] and safety specific [e.g., 6–8], have been found to be associated with employee workplace safety-related behaviors, and safety-related behaviors have been identified as important antecedents of workplace accidents and injuries [9]. There is variation in how site managers lead construction work, with some leadership behaviors apparently promoting safety while others apparently impede it [10–12].

A recent observational study among construction-site managers in Denmark and Sweden indicate contingent reward (CR) behaviors to be the most common leadership behaviors on construction sites [11]. CR leadership behaviors can be defined as the degree to which there are constructive exchanges between leaders and employees in which the leader clarifies expectations and what rewards can be expected when these expectations are met [13]. These behaviors also entail that the leader provides employees with material and/or psychological rewards when expectations are met [14]. CR leadership behaviors therefore also include monitoring employee performance and providing feedback to employees [e.g., 15, 16]. CR behaviors have been found to be associated with safety outcomes, such as safety climate, employee safety behaviors, and injury rates among employees [e.g., 5, 10, 15, 17, 18].

Considering the recognized problems of occupational accidents, injuries, and work-related diseases in the construction industry [1–4] and the potential positive impact of construction-site managers’ safety-leadership behaviors in mitigating these problems [e.g., 5–7, 10] it is important to investigate how leadership behaviors promoting safety on construction sites are fostered among construction-site managers.

One of the pathways through which construction-site managers’ safety leadership may influence workplace safety is through its influence on construction-site supervisors’ safety-leadership behaviors, via the mechanism of role modeling and imitation [19, 20]. Sims and Manz [19] argued that modeling and imitation occur regularly in organizations and are central to organizational functioning. Leader modeling may establish new behaviors and increase the frequency of existing ones [19]. In the construction industry, previous research has shown how construction-site managers can influence employee behavior through role modeling [12].

Performing the same behavior as other people in the same context is likely to be reinforced [21]. The theory of operant conditioning [21] stipulates that behaviors are primarily learnt in three steps: a) antecedent stimuli trigger a behavior; b) the behavior is performed; and c) consequences reinforce the behavior. Bandura and McDonald [22] demonstrated how the behavior of role models can be an important antecedent stimulus for follower behavior. However, exposure to such an antecedent stimulus is itself insufficient for role modeling (i.e., imitative/observational learning) to occur [23]; the behaviors of role models may trigger followers’ behaviors, but only if these behaviors are functional (i.e., result in positive consequences for followers) the behaviors are positively reinforced (i.e., learning occurs). However, when role models receive positive reinforcement and a potential learner observes this positive reinforcement, the learner is more likely to imitate the behavior [19, 24]. Hence, leadership behaviors that are functional (to the role model or the observer) are more likely to be learnt than are nonfunctional behaviors. It has been suggested that leaders can learn how to use CR behaviors more effectively by observing role models of these behaviors [16].

The overall purpose of this study was to investigate how safety-leadership practices can be developed within the construction industry, specifically focusing on the managerial role modeling of construction-site managers. We hypothesize that construction-site supervisors align their leadership behaviors with the leadership behaviors of their construction-site managers. In other words, site managers’ behaviors are expected to prospectively influence supervisors’ behaviors.

## 2 Methods

This study is part of the Safety-Leadership Project, a three-wave longitudinal study including three cohorts, investigating the importance of safety leadership in the Swedish construction industry. This study is based on data from the first two measurement waves, collected in May and August 2020 (cohort 1), October 2020 and February/March 2021 (cohort 2), and April/May 2021 and August 2021 (cohort 3), respectively. Ethics approval was obtained from the Swedish Ethical Review Authority (dnr. 2019-02471). Informed consent was obtained from the participants before inclusion in the study.

### 2.1 Procedure

The site managers who had agreed to participate in the Safety-Leadership Project were instructed to provide email addresses to all supervisors contracted to work on the respective construction sites for the entire duration of the research project. Electronic questionnaire surveys were sent via e-mail to all supervisors. Response rates were 66% in the first measurement and 52% in the second measurement. In total, 61 supervisors completed the questionnaire on both measurement occasions.

### 2.2 Participants

All supervisors who had provided self-report data and ratings of their site manager on both measurement occasions (in practice they had responded to 90% or more of the questionnaire items) were included in the analyses (*n* = 51, i.e., 84% of the original longitudinal sample). These supervisors were mostly men (86%) aged 23–68 years (*m* = 40.2, *sd* = 11.4). On average, they had spent 7.1 years working as supervisors (*sd* = 6.3, range: 0–30 years). The supervisors were nested within 29 different site managers and their tenure with their current site manager was 1.5 years on average (*sd* = 1.2, range: 0–5 years).

### 2.3 Measures

#### 2.3.1 Generic contingent reward behaviors

Generic CR behaviors were assessed using four items from Avolio and Bass’s [25] Multifactor Leadership Questionnaire. These items had seven fixed response alternatives ranging from “never” (1) to “always” (7). Respondents rated the behaviors of their site manager using these four items (sample item: “My site manager expresses satisfaction when I meet expectations”) and their own behaviors using the same four items (sample item: “I express satisfaction when others meet expectations”).

#### 2.3.2 Safety-specific contingent reward behaviors

Safety-specific CR behaviors were assessed using three items developed within the present research project. All these items had seven fixed response alternatives ranging from “never” (1) to “always” (7). Respondents rated both the behaviors of their site manager (sample item: “I receive positive feedback from my site manager when I tell him/her about safety risks at work”) and their own behaviors (sample item: “I provide positive feedback to my subordinates when they tell me about safety risks at work”). These items are reported in Table 1.

**Table 1:** Questionnaire items measuring safety-specific contingent reward behaviors

**Site manager behavior**
*I receive positive feedback from my site manager when* . . .
. . . I tell him/her about safety risks at work.
. . . I give suggestions on how to improve safety.
. . . he/she sees me perform a work task in accordance with safety rules and regulations.
**Supervisors’ own behavior**
*I provide positive feedback to my subordinates when* . . .
. . . they tell me about safety risks at work.
. . . they give suggestions on how to improve safety.
. . . I see them perform a work task in accordance with safety rules and regulations.

### 2.4 Statistical analysis

When investigating change over time, measurement (or factorial) invariance is a necessary prerequisite for valid inference and interpretation [26]. Or, in other words, when drawing conclusions based on longitudinal data, we need to first ensure that the same thing is being measured in the same way over time. Therefore, we first investigated factorial invariance (FI) over the two measurement occasions. This was done by testing a series of subsequent, increasingly stringent confirmatory factor analysis (CFA) models [27]. These subsequent models were constrained in accordance with configural FI (i.e., equality of the number and pattern of factors and factor loadings), weak/metric FI (i.e., also adding equality constraints on factor loadings), and strong/scalar FI (i.e., also adding equality constraints on indicator intercepts). To set the scale of the latent variables, the first factor loading of each latent variable was set to 1.0. Error terms were allowed to auto-correlate over time. Model fit was evaluated using a combination of fit indices: the χ^2^ statistic with its associated degrees of freedom and *p*-value, the comparative fit index (CFI), and the root mean square error of approximation (RMSEA) with its associated 90% confidence interval. These fit indices were interpreted in accordance with conventional cutoff criteria [see, e.g., 28]. For tests of FI, the delta χ^2^ criterion was used, that is, if the χ^2^ statistic suggested significantly worse model fit in relation to the degrees of freedom when adding constraints to a model, these constraints were considered not tenable. Supervisors’ ratings of their site managers and supervisors’ self-ratings were tested in two separate models. Fit indices for these models are reported in Tables 2 and 3.

**Table 2:** Fit indices for tests of factorial invariance (FI) over the two measurement occasions of the measurement/confirmatory factor analysis model, including supervisors’ ratings of *their site manager*’*s* generic contingent reward and safety-specific contingent reward behaviors (*n* = 51)

	χ^2^	CFI	RMSEA (90% CI)	Model comparisons	*Δ* >χ^2^
1. Configural FI	χ^2^ = 97.74, *df* = 64, *p* = 0.004	0.919	0.103 (0.059; 0.142)
2. Metric FI	χ^2^ = 101.54, *df* = 69, *p* = 0.007	0.922	0.097 (0.053; 0.136)	2 vs 1	*Δ* χ^2^ = 3.80, *Δ* *df* = 5, *p* = 0.58
3. Scalar FI	χ^2^ = 114.49, *df* = 76, *p* = 0.003	0.907	0.101 (0.060; 0.137)	3 vs 2	*Δ* χ^2^ = 12.95, *Δ* *df* = 7, *p* = 0.07

**Table 3:** Fit indices for tests of factorial invariance (FI) over the two measurement occasions of the measurement/confirmatory factor analysis model including supervisors’ ratings of *their own* generic contingent reward and safety-specific contingent reward behaviors (*n* = 51)

	χ^2^	CFI	RMSEA	Model comparisons	*Δ* χ^f2^
1. Configural FI	χ^2^ = 89.90, *df* = 64, *p* = 0.018	0.922	0.090 (0.039; 0.131)
2. Metric FI	χ^2^ = 94.62, *df* = 69, *p* = 0.022	0.923	0.086 (0.034; 0.127)	2 vs 1	*Δ* χ^2^ = 4.73, *Δ* *df* = 5, *p* = 0.45
3. Scalar FI	χ^2^ = 98.19, *df* = 76, *p* = 0.044	0.933	0.076 (0.013; 0.117)	3 vs 2	*Δ* χ^2^ = 3.56, *Δ* *df* = 7, *p* = 0.83

The model fit for the baseline models was considered acceptable, albeit with slightly low values for the CFI and slightly high values for the RMSEA (see Tables 2 and 3). The tests of FI indicate scalar FI over measurement occasions for both models, i.e., factor loadings and indicator intercepts did not significantly differ over time.

Thereafter, eight mean-level indexes were created such that high values indicate high levels of the respective behaviors: site manager generic CR behaviors at T1 (α= 0.78) and T2 (α= 0.80); site manager safety-specific CR behaviors at T1 (α= 0.86) and T2 (α= 0.85); self-reported generic CR behaviors at T1 (α= 0.76) and T2 (α= 0.76); and self-reported safety-specific CR behaviors at T1 (α= 0.71) and T2 (α= 0.87).

Finally, two cross-lagged panel models (CLPMs) were tested within a path model framework: one for generic CR behaviors and one for safety-specific CR behaviors. These models were fully saturated and included all potential cross-sectional (i.e., within-wave) correlations and all potential longitudinal regression weights (i.e., autoregressive as well as cross-lagged). This analytic approach enables us to investigate the prospective influence of site manager safety-leadership behaviors at time 1 on supervisor safety-leadership behaviors at time 2, while controlling for the effects of supervisor safety-leadership behaviors at time 1. It simultaneously enables us to investigate reversed effects (i.e., the effects of supervisor safety-leadership behaviors at time 1 on site manager safety-leadership behaviors at time 2 while controlling for the effects of site manager safety-leadership behaviors at time 1.) All models were tested using AMOS v. 28, employing the maximum likelihood estimator.

## 3 Results

Selected parameter estimates for the cross-lagged panel path models are presented for generic CR behaviors in Table 4 and for safety-specific CR behaviors in Table 5. Supervisors’ ratings of their site managers’ and their own generic CR behaviors were associated at T1 (*r* = 0.30, *p* = 0.04), while no such cross-sectional association was found for safety-specific CR behaviors (*r* = 0.12, *p* = 0.41).

**Table 4:** Selected parameters for the estimated cross-lagged panel (path) model investigating the relationship between supervisors’ ratings of their site manager’s and their own generic contingent reward (CR) behaviors (*n* = 51)

			Estimate	S.E.	*p*	Standardized estimate
***Correlations at T1***
Site manager’s generic CR behaviors	↔	Supervisors’ generic CR behaviors	0.22	0.11	0.04	0.30
***Autoregressive regression weights T1*** ⟶***T2***
Site manager’s generic CR behaviors T1	⟶	Site manager’s generic CR behaviors T2	0.73	0.09	<0.001	0.75
Supervisors’ generic CR behaviors T1	⟶	Supervisors’ generic CR behaviors T2	0.47	0.10	<0.001	0.51
***Cross-lagged regression weights T1*** ⟶ ***T2***
Site manager’s generic CR behaviors T1	⟶	Supervisors’ generic CR behaviors T2	0.24	0.09	0.01	0.29
Supervisors’ generic CR behaviors T1	⟶	Site manager’s generic CR behaviors T2	0.13	0.10	0.21	0.11

**Table 5:** Selected parameters for the estimated cross-lagged panel (path) model investigating the relationship between supervisors’ ratings of their site manager’s and their own safety-specific contingent reward (CR) behaviors (*n* = 51)

			Estimate	S.E.	*p*	Standardized estimate
***Correlations at T1***
Site manager’s safety-specific CR behaviors	↔	Supervisors’ safety-specific CR behaviors	0.10	0.12	0.41	0.12
***Autoregressive regression weights T1*** ⟶***T2***
Site manager’s safety-specific CR behaviors T1	⟶	Site manager’s safety-specific CR behaviors T2	0.46	0.12	<0.001	0.46
Supervisors’ safety-specific CR behaviors T1	⟶	Supervisors’ safety-specific CR behaviors T2	0.68	0.11	<0.001	0.63
***Cross-lagged regression weights T1*** ⟶ ***T2***
Site manager’s safety-specific CR behaviors T1	⟶	Supervisors’ safety-specific CR behaviors T2	0.18	0.09	0.04	0.22
Supervisors’ safety-specific CR behaviors T1	⟶	Site manager’s safety-specific CR behaviors T2	0.33	0.15	0.03	0.26

The autoregressive regression weights (i.e., stability coefficients) were moderate to high for both types of behaviors, and for both supervisors’ ratings of their site managers and for supervisors’ self-ratings: β= 0.75, *p* < 0.001 for site managers’ generic CR behaviors; β= 0.46, *p* < 0.001 for site managers’ safety-specific CR behaviors; β= 0.51, *p* < 0.001 for supervisors’ generic CR behaviors; and β= 0.63, *p* < 0.001 for supervisors’ safety-specific CR behaviors.

Supervisors’ ratings of their site managers’ safety-specific CR behaviors at T1 significantly influenced the supervisors’ self-rated safety-specific CR behaviors at T2 (β= 0.22, *p* = 0.04). Similar results were found for generic CR behaviors, i.e., site managers’ generic CR behaviors at T1 influenced supervisors’ generic CR behaviors at T2 (β= 0.29, *p* = 0.01). The hypothesis that site managers’ behaviors prospectively influence supervisors’ behaviors was therefore supported. Reversed effects of roughly the same magnitude were found for safety-specific CR behaviors, that is, supervisors’ behaviors at T1 (self-rated) influenced site managers’ behaviors at T2 (also rated by the supervisors) (β= 0.26, *p* = 0.03). However, no reversed effect was found for generic CR behaviors.

## 4 Discussion

The present results indicate a prospective effect of construction-site managers’ generic and safety-specific CR behaviors on their supervisors’ generic and safety-specific CR behaviors over the study period of approximately four months. This finding could be interpreted to mean that the construction-site managers function as role models for construction-site supervisors’ safety leadership. This finding concurs with previous research that indicates managerial role modeling to be an important mechanism for the socialization of organizational behaviors [20]. The effect of site managers’ safety leadership on construction site safety outcomes suggested in previous cross-sectional research [e.g., 10] may therefore be both direct (i.e., site managers’ leadership behaviors directly influence employee safety behaviors) and indirect through its effect on subordinate supervisors (i.e., site managers’ safety-leadership behaviors influence subordinate supervisors’ leadership behaviors, which in turn influence employee safety behavior). The link between construction-site supervisors’ leadership behaviors and employee safety behaviors has been outlined in previous cross-sectional research [e.g., 5–7].

Yukl [29] argued that leadership entails influencing and facilitating individual and collective efforts to meet shared organizational objectives, and that this can be accomplished by influencing processes that determine performance. Christian et al. [9] argued that leaders’ safety priorities provide a basis for how safety climate is perceived by employees, which in turn influences their safety behaviors. CR leadership practices may thus in part increase workplace safety through highlighting and reinforcing the organizational contingencies for safety. It has been suggested that managers’ CR behaviors are among the key factors explaining the effects of an organizational meritocratic system on individual job performance [29]. More specifically, in the context of individual pay for performance, Han et al. [30] suggested that the effect of individual pay for performance on individual job performance is mediated by performance-reward expectancy, and that this mediated effect is larger among employees whose managers display a high degree of CR behaviors. It seems reasonable to assume that such a mechanism would also apply to safety-specific outcomes, i.e., construction-site managers’ and supervisors’ CR behaviors impact the effect of an organizational reward system prioritizing safe outcomes on the safety-related behaviors of the employees.

The present results also indicate a prospective effect of supervisors’ safety-specific CR behaviors on their site managers’ safety-specific CR behaviors. This finding indicates that role modeling on construction sites is not only a top-down process, but is simultaneously also a bottom-up process. In other words, site managers act as role models for supervisors when it comes to developing safety-leadership behaviors on the construction site, but site managers are also influenced by their subordinate supervisors’ safety-leadership behaviors. There seems to be reciprocal interaction between site managers and supervisors in which they influence each other, and together shape safety-leadership practices on the construction site.

This study focused on the role modeling of leadership behaviors that previous research has identified as positively influencing workplace safety. It should be noted, however, that the mechanism of role modeling also can apply to unsafe leadership practices. Previous research has illustrated how site managers can act as role models of both safe (e.g., using personal protection equipment) and unsafe (e.g., walking on unsafe scaffolds) behaviors [12].

### 4.1 Strengths and limitations

This study focuses on managerial role modeling as a potential mechanism for improving workplace health and safety in the construction industry. An important strength of this study is its longitudinal design, which allows us to investigate prospective relationships, including potential reversed and reciprocal effects.

All data included in the present analyses were obtained from a single source (i.e., the supervisors) using a single method of data collection (i.e., questionnaire data). This may lead to common-method variance, potentially inflating the associations between the study variables. This potential problem could be mitigated by including, for example, site manager self-ratings or ratings made by an independent observer. On the other hand, when investigating the potential effects of role modeling, focusing on site manager leadership behaviors *as perceived and understood by the subordinate supervisor*, subordinate supervisor ratings of these behaviors would seem to be the most relevant source of information.

Another limitation is the small sample size, which leads to reduced power both directly and indirectly through the limitations this imposes on our ability to account for indicator unreliability (e.g., by modeling latent variables in the CLPMs). It should also be noted that using small sample sizes may lead to biased results. The present analyses were conducted using maximum likelihood estimation. This type of estimator relies on asymptotic theory, meaning that estimates can be expected to be unbiased if sample sizes are sufficiently large [see, e.g., 31,32]. However, with small to moderate sample sizes, such large-sample properties may not apply.

The small sample size also makes it difficult to test more complex models, for example, including other potentially important predictor and/or moderator variables. Bandura [23] suggested that factors such as motivation and anticipation of positive and negative reinforcement may influence imitative learning. Leadership research indicates that the role modeling mechanism may be influenced by observer characteristics such as self-esteem, as well as by observer-perceived role model characteristics such as success and competence [20]. Research focusing on identifying contextual factors that influence supervisors’ engagement in safety leadership indicates that social support and perceived autonomy may be two aspects that promote engagement, while role overload and production demands may hinder engagement [33]. Such contextual factors may also affect the role modeling mechanism investigated in the present study.

Furthermore, the small sample size means that it is not feasible to control for the nested structure of the data (i.e., supervisors being nested within site managers). This may lead to underestimated standard errors and, hence, an increased risk of making a type-1 error [see, e.g., 34]. However, this problem is probably less severe in designs such as the present one, which includes smaller groups (i.e., only a few supervisors nested within each site manager).

The range of seniority of the examined supervisors was 0–30 years, and they had 0–5 years of tenure with their current site manager. Both these factors could be expected to influence the strength of the role modeling effect (i.e., moderating the effect of site manager leadership behaviors on supervisor leadership behaviors). If role modeling is a mechanism for the socialization of organizational behavior [20], then we would expect the effect of role modeling to be stronger among supervisors with relatively little experience of working as supervisors and with shorter tenure in their present organization. At the same time, we would expect the role modeling effect to be stronger among supervisors having worked with, and thereby having observed, their site manager for a longer period. Furthermore, given the complex nature of the construction industry, supervisors can work with several other potential role models, who, simultaneously or consecutively, may exert a role modeling influence that we cannot detect here. If different role models display divergent safety-leadership behaviors, the role modeling effect of any single one of them may be reduced, leading to effect masking.

## 5 Conclusions

The results of the present study indicate that construction-site managers’ safety-leadership behaviors may improve workplace safety through their effects on construction-site supervisors’ safety-leadership behaviors, via the mechanism of role modeling. This finding implies that site managers can use role-modeling as a way of influencing the leadership behaviors of their subordinate supervisors and thereby the safety performance of their construction sites. The results further indicate that role modeling on construction sites is not only a top-down process, but is simultaneously also a bottom-up process. In other words, site managers act as role models for supervisors when it comes to developing safety-leadership behaviors, but site supervisors’ behaviors may also influence the safety-leadership behaviors of their site managers. In sum, these results highlight how all leaders on construction sites are important for workplace safety and should be included in developing the safety-leadership practices in the construction industry.

## Ethical approval

Ethics approval was obtained from the Swedish Ethical Review Authority (dnr. 2019-02471).

## Informed consent

Informed consent was obtained from the participants before inclusion in the study.

## Conflict of interest

The authors declare that they have no conflict of interest.
